# Length Redundancy and Twist Improve the Biomechanical Properties of Polytetrafluoroethylene Bypass Grafts

**DOI:** 10.1016/j.avsg.2019.04.007

**Published:** 2019-11

**Authors:** Regent Lee, Miranda Stoddart, Igor Dyson, Ismail Cassimjee, Ashok Handa, Christopher P. Cheng

**Affiliations:** 1 Nuffield Department of Surgical Sciences, John Radcliffe Hospital, University of Oxford, Oxford, Oxfordshire, UK; 2 Department of Engineering Science, University of Oxford, Oxford, Oxfordshire, UK; 3 Department of Vascular Surgery, Stanford University, Palo Alto, CA

## Abstract

**Background:**

The iliofemoropopliteal artery significantly changes path length during normal hip and knee flexion. Prosthetic bypass grafts, such as polytetrafluoroethylene (PTFE) grafts, are relatively stiff and thus can subject graft anastomoses to high tension when the path length increases. The aim of this study was to examine the influence of length redundancy and twist on the biomechanical properties of PTFE bypass grafts.

**Methods:**

Unreinforced and ring-reinforced PTFE grafts were loaded in an axial mechanical testing machine to measure the tensile and compressive axial forces with varying levels of length redundancy and axial twist.

**Results:**

Adding 5-15% length redundancy to a graft decreases the force to cause 5% extension by > 90% without substantially increasing shortening forces. Adding 4.5°/cm of axial twist imparts a corkscrew shape to the graft without increasing extension or shortening forces in the presence of length redundancy. Ring-reinforced PTFE grafts require more length redundancy to experience these reductions in forces especially in the presence of axial twist.

**Conclusions:**

A modest amount of length redundancy and twist (i.e., a cork-screw condition) confers improved biomechanical properties in a PTFE graft, especially in ring-reinforced grafts. This should be taken into consideration when fashioning an arterial bypass graft in the iliofemoropopliteal segment.

## Introduction

The iliofemoropopliteal artery significantly changes conformation and path length during normal hip and knee flexion because of the iliofemoral artery being anterior to the hip joint and the popliteal artery being posterior to the knee joint.^1^, ^2^ Changes in the axial length also vary considerably in the population because of variations in vascular anatomy and pathology.^3^, ^4^, ^5^ These observations, along with the fact that vascular grafts tend to be relatively stiff in the longitudinal direction, have important implications and pose a conundrum during lower limb bypass grafting. Given the abovementioned considerations, it is intuitive to allow some redundant length in the graft to accommodate for the anticipated change in the axial length during every day hip and knee flexion. However, excess length of a bypass graft is commonly thought to predispose to graft kinking and result in bypass graft failure.^6^ These conflicting factors lead to inconsistent surgical techniques and may affect the outcomes of infrainguinal bypass grafts.

Prosthetic bypass grafts, such as polytetrafluoroethylene (PTFE) grafts, are relatively stiff and manufactured as straight tubular structures. However, a growing body of evidence suggests corkscrew/spiral-shaped vascular conduits improve the hemodynamics of blood flow.^7^, ^8^, ^9^, ^10^ In addition, much product development and regulatory progress has been made with this design concept.^11^, ^12^, ^13^, ^14^ These emerging literature call for a rethink in the approach to fashioning infrainguinal bypass grafts.

The aim of this study is to examine the influence of length redundancy and twist on the biomechanical properties of PTFE bypass grafts. We hypothesize that redundancy of graft length reduces the tensile forces on the anastomoses of a vascular graft in the lengthening direction without sacrificing compressive properties in the shortening direction. We further hypothesize that a slightly twisted (corkscrew-shaped) graft may be able to accommodate length change more smoothly, thus improving the mechanical properties of the bypass graft while conveniently improving hemodynamics.

## Methods and Results

**Fig. 1:**
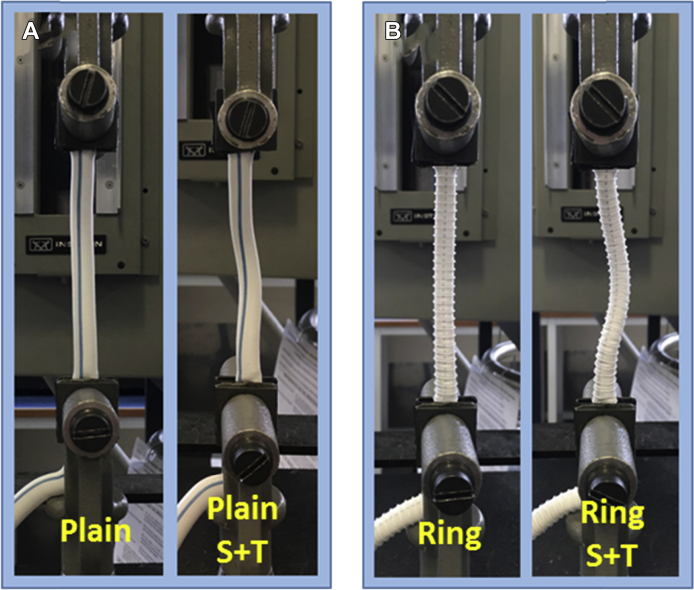
PTFE grafts are loaded in an Instron tester to assess the mechanical properties during different conditions: A PTFE graft of 8 mm diameter was set in the “Plain” (100 mm graft with 100 mm distance between grips, no twist) and “Plain S + T” (110 mm graft with 100 mm distance between grips (10% slack), 4.5°/cm twist) conditions **(A)**. A ring-reinforced graft was set in the “Ring” (100 mm graft with 100 mm distance between grips, no twist) and “Ring S + T” (110 mm graft with 100 mm distance between grips (10% slack), 4.5°/cm twist) conditions **(B)**. A metal rod was placed in the lumen of the graft so that the cross-section of the graft remained circular when gripped between jaws.

We chose representative types of PTFE grafts with 8-mm diameter for the experiments. Plain and ring-reinforced (Atrium Hybrid PTFE vascular graft) vascular surgical grafts were used for axial extension and shortening testing on an “Instron” mechanical testing machine (Model #5582 with a 100N load cell) (Fig. 1). For each graft, the short, gripped length of each end was plugged by a metal mandrel so that the ends could be gripped between jaws and remain circular in cross-section. Each graft was cyclically preconditioned for several cycles and then axially extended by 8%, brought back to baseline, axially shortened by 25%, and finally brought back to baseline, all in one uninterrupted cycle. While the speed of femoral artery path length change is of the order of ∼30 cm/sec (as estimated from 100 steps/min * 10 cm length change/step * 2 length changes/step), the rate of applied axial translation speed was 5 mm/sec for both directions, constituting a quasistatic loading. Samples were tested in the taut and untwisted (100 mm graft with 100 mm distance between grips, no twist; “Plain” and “Ring”) and 10% slack and 45° twist (110 mm graft with 100 mm distance between grips, 4.5°/cm twist; “Plain S + T” and “Ring S + T”) conditions.

**Fig. 2:**
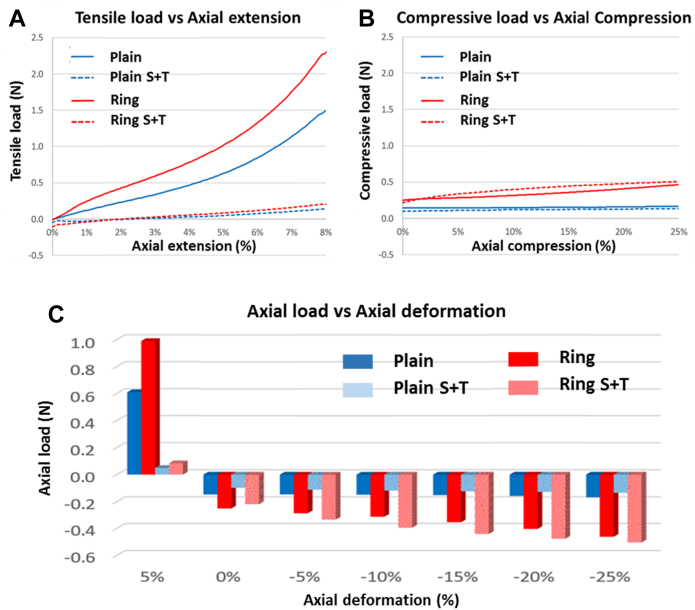
The tensile, compressive, and axial loads at different axial length. Each graft was axially extended by 8%, brought back to baseline, and then axially shortened by 25%. The axial translation speed was 5 mm/sec for both directions to test for: tensile load versus axial extension **(A)** and compressive load versus axial compression **(B)** at various force settings. The same graft conditions were used to assess the axial loads at 5% extension, and 0%, 5%, 10%, 15%, 20%, and 25% shortening **(C)**. N, Newtons; “Plain,” 100 mm graft with 100 mm distance between grips, no twist; “Plain S + T,” 110 mm graft with 100 mm distance between grips (10% slack), 4.5°/cm twist; “Ring,” 100 mm graft with 100 mm distance between grips, no twist; “Ring S + T,” 110 mm graft with 100 mm distance between grips (10% slack), 4.5°/cm twist.

We further tested the tensile load versus axial extension and compressive load versus axial compression for the plain and ring-reinforced grafts, both while taut and untwisted, and with 10% slack and 45° twist (Fig. 2). This shows that for axial extension (Fig. 2A), adding 10% slack and 45° twist significantly decreases the amount of tensile force required to lengthen both the plain and ring-reinforced grafts. In the taut and untwisted configuration, the ring-reinforced grafts require substantially greater tensile force for extension than the plain grafts. For compression (Fig. 2B), the addition of 10% slack and 45° twist yields very little reduction in compressive force to axially shorten the graft. Similar to the extension direction, the ring-reinforced grafts require greater compressive force to be axially shortened than the plain grafts. We then tested the grafts while taut and untwisted, and with 10% slack and 45° twist, at states of 5% extension, and 0%, 5%, 10%, 15%, 20%, and 25% shortening between the two grips (Fig. 2C). In extension, the presence of 10% slack and 45° twist dramatically reduces axial tensile loads during 5% extension. In compression, the addition of 10% slack and 45° twist hardly changes the axial compressive loads for 0%, 5%, 10%, 15%, 20%, and 25% shortening between the two grips. There is, however, a gradual increase in compressive load with greater amounts of axial shortening. Note that the ring-reinforced graft generates greater loads, in both the tensile and compression directions, than the plain graft.

We measured the changes in axial load required to cause 5% extension and 5% and 10% shortening in a graft with the addition of various levels of slack as compared with the taut and untwisted configuration (Table IA). This data shows that adding slack decreases the axial load needed to cause 5% extension by approximately 0.75 N (∼75 grams of weight) and 1.2 N (∼120 grams of weight) for the plain and ring-reinforced grafts, respectively. The amount of slack, ranging from 5 to 15%, does not appear to affect the reduction in axial load. Note that the axial load needed to shorten the graft is relatively less affected by the addition of slack.

**Table I:** Axial load (Newtons) required to cause 5% extension and 5% and 10% shortening in a graft with the addition of various levels of slack as compared with the taut and untwisted configuration (A); with the addition of 45° of twist (4.5°/cm) as compared with the untwisted configuration (B); with the addition of 10% slack and 45° of twist (4.5°/cm) as compared with the untwisted configuration with 0%, 5%, 10%, and 15% slack (C)

A	Change in axial load required (Newton)					
	Plain PTFE Graft			Ring-reinforced PTFE Graft		
	5% slack	10% slack	15% slack	5% slack	10% slack	15% slack
5%	−0.76	−0.76	−0.76	−1.24	−1.27	−1.30
−5%	0.00	−0.01	−0.01	−0.03	−0.07	−0.12
−10%	0.00	−0.01	−0.02	−0.04	−0.09	−0.15

B	Change in axial load required (Newton)					
	Plain PTFE Graft			Ring-reinforced PTFE Graft		
	5% slack	10% slack	15% slack	5% slack	10% slack	15% slack
5%	NA	0.19	0.05	NA	0.37	0.10
−5%	0.05	0.04	0.04	0.10	0.02	0.01
−10%	0.04	0.04	0.04	0.02	0.01	0.02

C	Change in axial load required (Newton)							
	Plain PTFE Graft				Ring-reinforced PTFE Graft			
	0% slack	5% slack	10% slack	15% slack	0% slack	5% slack	10% slack	15% slack
5%	−0.56	0.05	0.03	0.03	−0.91	0.03	−0.05	−0.08
−5%	0.03	0.03	0.03	0.03	−0.05	−0.08	−0.09	−0.07
−10%	0.03	0.03	0.03	0.03	−0.08	−0.09	−0.07	−0.04

We further measured the changes in axial load required to cause 5% extension and 5% and 10% shortening in a graft with the addition of 45° of twist (4.5°/cm) as compared with the untwisted configuration (Table IB). There are increases in axial load required to extend the graft with the addition of twist, with greater increases in situations of lower amounts of slack. The force required to shorten the path length of the graft is relatively unaffected by twist, however, twist causes slightly greater axial loads.

We assessed the axial load required to cause 5% extension and 5% and 10% shortening in a graft with the addition of 10% slack and 45° of twist (4.5°/cm) as compared with the untwisted configuration with 0%, 5%, 10%, and 15% slack (Table IC). There is a major reduction of tensile force needed to extend the grafts with the addition of 10% slack and 45° of twist only for the 0% slack scenario (−0.56 N for plain, −0.91 N for ring-reinforced). All other scenarios experience relatively low changes in axial load requirements. In general, there is a small addition of axial load required to deform the plain graft whereas there is a small reduction of axial load required to deform the ring-reinforced graft due to superposing 10% slack and 45° of twist.

## Discussion

The addition of varying amounts of graft length redundancy (axial slack) significantly affected the forces generated during extension and shortening testing of plain and ring-reinforced vascular grafts. When either graft design is taut, extension of even 5% requires substantial tensile force due to the relatively high stiffness in the graft material. This is exacerbated in the ring-reinforced design because ring reinforcement augments effective stiffness. Adding redundancy to the graft length ameliorates this problem by practically eliminating the required tensile force to lengthen the graft path length (reduction of >90%). This happens because the graft material is not significantly strained.

Interestingly, this dramatic reduction in tensile lengthening force is not sensitive to the amount of graft redundancy as long as the graft never exceeds the taut state. Slack ranging from 5 to 15% does not appear to affect the reduction in axial load. Because ring-reinforced grafts require more force to deform, they exhibit greater reduction in axial load from additional slack than plain grafts. In addition, the extra graft redundancy is not at all detrimental to compressive forces needed to shorten the graft path length. This is because once the graft is past the point of slack and has buckled, further buckling requires relatively little additional force. Note that the ring-reinforced graft requires more compressive force to shorten the graft path length than the plain graft because the structure of the ring-reinforced graft causes higher effective stiffness.

While adding redundancy to the graft length is highly effective at reducing tensile forces during lengthening and not detrimental to compressive forces during shortening, adding axial twist to the graft appears to cause increased tensile forces during path lengthening in the presence of low redundancy. Twist rotates material fibers away from being parallel to the axis of the graft, whereas axial extension requires these angled fibers to be realigned toward being parallel to this axis. This realignment requires shear strain between these angled fibers and thus additional axial tension. This is especially apparent in ring-reinforced grafts, which is consistent with their higher effective stiffness. Conversely, the presence of axial twist does not appear to substantially impact the required compressive force to shorten the path length of a graft.

The knee is usually placed in a flexed position during the performance of a below knee popliteal bypass graft anastomosis to allow adequate exposure through a medial approach. It is routine practice to allow some redundancy in the graft in anticipation of the additional length required when the knee is fully extended. Our results suggest adding additional amount of slack to a vascular graft in vivo could dramatically reduce tensile loads on the anastomotic sites during path lengthening. For example, during hip and knee extension, the straight path length of the iliofemoropopliteal vessels can increase by 10% or more, so that adding slack to the graft may benefit.^4^, ^15^, ^16^, ^17^ The ∼1 N of repetitive tension caused even by 5% graft extension can be detrimental to the long-term durability of an anastomosis stability. Conveniently, this tensile load benefit is not sensitive to the exact amount of slack (in this study 5-15% slack was tested), so attention should be paid to providing enough slack with a little extra margin. This is especially important in the case of ring-reinforced grafts, which are designed to prevent kinking to maintain circular lumens, where slack is necessary to accommodate axial twist without causing increased tensile force on the graft anastomoses. As an added bonus, simultaneous slack and twist leads to a corkscrew shape of the graft, potentially improving hemodynamics. In addition, as up to 4.5°/cm of twist (equivalent to 90° of twist over a 20 cm graft length) did not cause appreciable changes in axial loads in the presence of sufficient slack, the amount of twist does not need to be very precise.

In this bench experiment design, we did not simulate the pulsatile blood flow and support of surrounding tissue which may prevent kinks in the graft. The axial force measurements were performed in a quasistatic state, meaning that: (1) while the forces may not be representative of actual in vivo forces, the results are instructive for any type of graft material/design because we eliminated inertial effects and (2) the results underestimate in vivo forces as vascular graft material is generally strain-rate hardening, so the importance of providing slack in a vascular graft may be heightened. We chose only two types of grafts and one diameter (8 mm) as representations of the synthetic graft types commonly used for femoral popliteal bypass. Further work will be required to demonstrate the external validity of our observations in vivo.

## Conclusion

A modest amount of length redundancy and twist (i.e. a cork-screwed condition) confers improved biomechanical properties in a PTFE graft. This should be taken into consideration when fashioning an arterial bypass graft in the iliofemoropopliteal segment. Alternatively, a highly axially compliant graft would accommodate the dynamics of the leg.
